# Global Longitudinal Strain as an Efficient Prognostic Tool in Hypertrophic Cardiomyopathy With Preserved Left Ventricular Ejection Fraction

**DOI:** 10.7759/cureus.30573

**Published:** 2022-10-22

**Authors:** Owen Battel, Kevin Newsome, Guillermo Izquierdo-Pretel

**Affiliations:** 1 Internal Medicine, Florida International University, Herbert Wertheim College of Medicine, Miami, USA

**Keywords:** ejection fraction, hypertrophic cardiomyopathy, speckle tracking, myocardial strain, speckle echo

## Abstract

Intending to demonstrate the utility of the global longitudinal strain (GLS) in patients with hypertrophic cardiomyopathy (HCM) and preserved ejection fraction, we describe a case of a 54-year-old female who presented to our emergency department with an acute onset of palpitations and chest pain. The patient was noted to have a new onset of atrial fibrillation. An echocardiogram showed an unimpaired ejection fraction suggesting normal left ventricular systolic function with findings of concentric left ventricular and apical hypertrophy. However, the speckled ultrasound revealed a GLS of -6.2%, suggesting marked impairment of ventricular wall movement. A CHA₂DS₂-VASc score was calculated and yielded a score of 2, indicating a moderate risk for thromboembolism. After a full evaluation of the case, the patient was started on anticoagulation due to her GLS result that suggested stasis within the left ventricle that may lead to a thrombus. Additional advice was to follow up closely for possible automatic implantable cardioverter defibrillator placement. We conclude that GLS is a cheap and easy tool to utilize as an additional prognostic marker for cardiovascular complications, particularly among those who have progression of HCM or start with symptoms such as atrial fibrillation and may have impaired left ventricular systolic function despite a preserved ejection fraction. Thus, we encourage the medical community to utilize and further study this novel technology.

## Introduction

Non-invasive evaluation of left ventricular (LV) systolic function by echocardiography has historically been quantified utilizing LV ejection fraction (LVEF); however, it is subject to intrinsic limitations [1]. Myocardial strain assessed using speckle tracking echocardiography has recently emerged as a marker for LV systolic function that may avoid the limitations of LVEF by measuring myocardial strain in longitudinal, radial, and circumferential directions regardless of the angle of the ultrasound probe. Speckle tracking analyzes the motion of speckles on ultrasound by tracking displacement during the cardiac cycle, allowing for the calculation of strain and strain rate [2]. 

Global longitudinal strain (GLS) is the most widely studied strain used to characterize LV systolic function [3]. LV GLS is capable of measuring the actual deformation of the subendocardial myocardium, which is very susceptible to ischemic changes, and boasts a superior sensitivity, specificity, positive predictive value, and negative predictive value when compared to electrocardiographic changes suggestive of ischemia [3]. The echocardiogram is conducted from the apical position and captures the standard four-chamber, two-chamber, and long-axis views. Six myocardial segments are analyzed for each view and the average strain value is obtained for each segment, totaling 18 segments between the three views. GLS is then calculated by taking the average of all peak systolic strain values [4]. 

Hypertrophic cardiomyopathy (HCM) may differ in clinical presentation, morphology, and prognosis. The annual risk of cardiac death in those with HCM is 5% and the presence of atrial fibrillation (AF), evidence of myocardial infarction on ECG, use of digoxin and diuretics, and a high New York Heart Association functional class at presentation are all associated with further decreased survival [5]. Independently, AF is the most common arrhythmia in adults, and many AF patients present with symptoms despite unimpaired LVEF. A previous study demonstrated that AF patients had more impaired GLS compared to patients without AF, despite similar LVEF measurements, suggesting that GLS may be used to identify impaired LV wall motion in these patients [6]. This case illustrates that GLS may be used as a marker of prognosis in cases where uncertainty is present, specifically in the progression of HCM associated with AF where a preserved LVEF in the echocardiogram is documented.

## Case presentation

**Figure 1:**
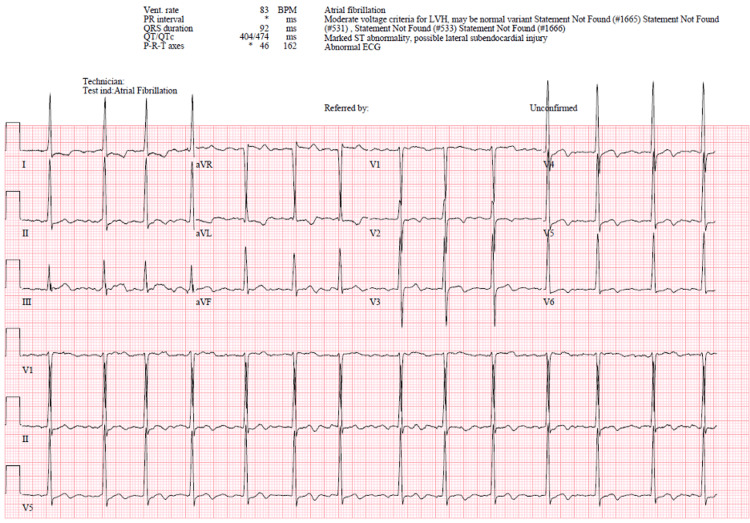
Twelve-Lead Electrocardiogram Demonstrating Atrial Fibrillation

A 54-year-old female with a history of hypertension and LV hypertrophy presented to our emergency department (ED) in Miami, Florida due to palpitations, chest pain, and weakness. The patient was traveling to Miami from Europe for a medical conference and has a history of episodes of supraventricular tachycardia (SVT) that she is able to control with atenolol 25 mg daily and carotid massage when symptoms arise. The patient admits to having a half-glass of champagne and half-glass of white wine at the concluding ceremony of the conference just prior to the onset of symptoms when she was notified by her apple watch that she was in AF, which prompted her to seek medical assistance. Upon arrival at the ED, the patient was confirmed to have sudden-onset AF by 12-lead EKG with a heart rate as high as 180 beats per minute (bpm) (Figure 1). The patient denied any history of AF, thyroid abnormalities, atherosclerotic disease, diabetes mellitus, as well as tobacco or recreational drug use. The patient reported a family history of HCM in her father and sudden cardiac death in her uncle. 

**Figure 2:**
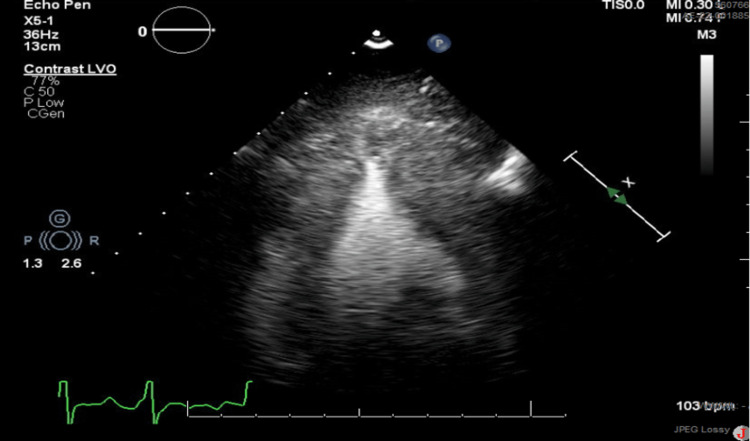
Echocardiography of the Patient’s Heart Left ventricular echocardiography shows prominent apical hypertrophy and narrowing of the left ventricular cavity, resulting in a “spade” morphology.

**Figure 3:**
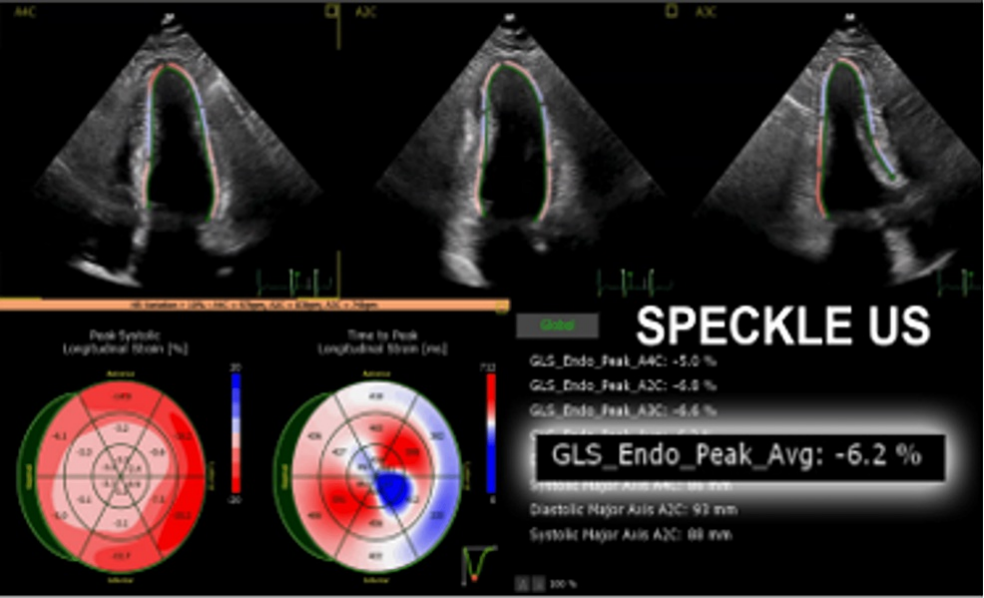
Speckle Echocardiography of the Patient’s Heart Left ventricular speckle echocardiography shows a decreased GLS of -6.2%. GLS, global longitudinal strain.

The patient was able to reproduce the results of previous angiograms, cardiac MRI, and echocardiogram on her smartphone, all of which were conducted in Europe due to her history of SVT and hypertension and reported normal cardiac function. Physical examination revealed a well-appearing woman in no acute distress with an irregularly irregular rhythm, no murmur, no gallop, and good pulses equal in all extremities except the right lower extremity with a near-absent pedal pulse. Clinical examination showed a blood pressure of 143/91 and a pulse of 106. Labs revealed normal troponin of 0.027. The echocardiogram showed moderate concentric and apical LV hypertrophy with normal LV systolic function with an ejection fraction of 60-65% (Figure 2). Speckled ultrasound, however, demonstrated a GLS of -6.2% suggesting marked impairment of ventricular wall movement (Figure 3). The patient had a return flight back to Europe scheduled for later the same evening and indicated that she had no intention of rescheduling it. Thus, time was of the essence when evaluating the patient and creating a treatment plan.

When evaluating whether the patient required preemptive anticoagulation due to the acute onset of AF, a CHA₂DS₂-VASc score was calculated and yielded a value of 2, indicating moderate risk for thromboembolism. In combination with the patient's impaired GLS, suggesting impaired LV wall motion and possible stasis, and upcoming transatlantic flight, she was started on Xarelto 20 mg daily for anticoagulation and advised to follow up with her cardiologist at home to evaluate for automatic implantable cardioverter defibrillator placement. By utilizing GLS, the patient’s abnormal value suggested that her acute-onset AF was due to the natural progression of HCM leading to arrhythmias, rather than an independent problem, and signified for the need for intervention.

## Discussion

GLS based on two-dimensional (2D) speckle tracking can detect more subtle changes in LV function than that is observable with LVEF [7]. This is thought to be due to the fact that longitudinal fibers in the subendocardium are most susceptible to pathological change [7]. Speckle tracking strain imaging provides an evaluation of LV systolic function in radial, longitudinal, circumferential, and rotation directions, with longitudinal strain measurements being the most reproducible of these [7]. There is abundant evidence documenting the diagnostic and prognostic value of GLS; however, the parameters have not been well defined. D’Elia et al. conducted a meta-analysis, which reported that regardless of clinical covariates, such as weight, age, and systolic blood pressure, a GLS value of <16% indicates significant myocardial dysfunction [8]. Similarly, Tower-Rader et al. noted that the majority of studies found an association between abnormal GLS results and increased cardiac events in patients with HCM during their systematic review [9]. 

AF is characterized by an inability of the atria to efficiently contract, leading to impaired ventricular filling and impaired LV systolic function. Thus, AF is an important determinant of LV function and might impair GLS even when LVEF is maintained [10]. HCM remains the most common hereditary cardiomyopathy and is commonly associated with the sustained arrhythmia of AF. Management of patients with AF includes symptomatic control, via rate and/or rhythm control, and prevention of adverse events such as thromboembolism, via anticoagulation [10]. Two studies published by Modin et al. and Dons et al. found that GLS was an independent predictor of mortality after multivariable adjustment in patients with AF while LVEF was not significantly associated with mortality [11,12]. Furthermore, a study published by Huang et al. found that GLS was an effective predictor of thrombogenesis in patients with AF [13]. The findings from these studies suggest that while LVEF may be unimpaired in patients with AF, there may still be LV dysfunction that may lead to thrombogenesis and impact patient mortality which is detectable with the measurement of GLS using speckled ultrasonography. 

Multiple studies have noted an association between impaired strain seen with GLS and histopathologic changes and myocardial fibrosis in patients with HCM and AF. In addition, studies are suggesting that GLS is more sensitive to detect fibrosis than cardiac MRI enhanced by gadolinium, but the application of GLS in the clinical management of patients with HCM and AF is still exploratory at this time due to a lack of consensus regarding what value of GLS should prompt a physician to alter care [9]. In a prospective study of 400 patients with HCM, Hongyun Liu et al. found that patients with GLS >-16% had significantly increased cardiac composite events than those with GLS <-16% [14]. The majority of patients with HCM have a normal or hyperdynamic systolic function, so even though the systolic function is generally predictive of outcomes in patients with heart failure, it is not a prognostic indicator for those with HCM. Despite normal systolic function, fibrosis and disruptions of normal cellular physiology indicate that there is a myopathy occurring [14]. 

Our patient with a history of AF and HCM presented with an acceptable LVEF of 60-65% on echocardiogram, but a greatly diminished GLS value of -6.2% and a concerning family history of adverse cardiac events. Based on the current evidence demonstrating the prognostic guidance of GLS as a predictor of thrombogenesis and mortality in AF patients and our clinical intuition, we came to the conclusion that treatment with pharmacologic anticoagulation is beneficial in our patient before a transatlantic flight.

## Conclusions

GLS measured using speckle tracking echocardiography shows promise as a marker for quantifying LV systolic function and the technology overcomes many of the intrinsic limitations of LVEF, the current gold standard. Studies have demonstrated that GLS is an independent marker for thrombogenesis and prognosis in patients with AF and preserved LVEF. GLS is an inexpensive and efficient marker that can be measured simultaneously with LVEF using the same echocardiography machines already deployed within hospitals. By providing an additional comprehensive marker of LV systolic function, clinicians will be better equipped to accurately evaluate patients with impaired systolic function, particularly in those with HCM, and associated AF who often have preserved LVEFs. Additional research on GLS is needed to optimally diagnose and risk-stratify patients with impaired LV systolic function to provide appropriate therapeutic care and ultimately improve patient outcomes. 
